# Probing boundary conditions of Productive Failure and analyzing the role of young students’ collaboration

**DOI:** 10.1038/s41539-019-0041-5

**Published:** 2019-03-26

**Authors:** Claudia Mazziotti, Nikol Rummel, Anne Deiglmayr, Katharina Loibl

**Affiliations:** 1 Institute of Educational Research, Ruhr-Universität Bochum Universitätsstr 150, 44801 Bochum, Germany; 2 Research on Learning and Instruction ETH Zurich, Clausiusstr. 59, 8092 Zurich, Switzerland; 3 Institute of Education, University of Education Freiburg, Kunzenweg 21, 79117 Freiburg, Germany

## Abstract

Productive Failure (PF) facilitates students’ conceptual knowledge by delaying instruction until after problem solving. While PF is well investigated in middle and high school students, little is known about its effectiveness in younger students. Studies in younger samples, which implemented delayed instruction designs similar to those used in PF studies, showed mixed results. However, these studies did not implement two core design components of PF: (1) contrasting and comparing student-generated solutions and the canonical solution during the instructional phase (contrasting activity), and (2) students’ collaboration in small groups during the initial problem-solving phase. Both components can be expected to contribute to the effectiveness of PF. In a quasi-experimental study with 228 fifth graders, we implemented the first component (contrasting activity) with all students to establish whether under this condition, problem solving prior to instruction would be more effective for younger students’ conceptual knowledge acquisition than direct instruction (i.e., problem solving after instruction). Further, we experimentally tested the effect of the second component (collaborative vs. individual problem solving) on students’ conceptual knowledge and the number of solution ideas generated during initial problem solving. We found no empirical support for either of our hypotheses. To explore the extent to which students’ collaboration actually achieved its potential and relates to students’ conceptual knowledge and solution ideas in PF, we conducted analyses of collaborative processes. Our study adds to the mixed results regarding the superiority of problem solving prior to instruction for young students, thus opening the discussion about age-related prerequisites as boundary conditions for PF.

## Productive Failure: young students don’t learn productively with Productive Failure

The beneficial effect of Productive Failure does not usually transfer to students who are younger than the typical age group. Productive Failure proposes that students gain more understanding if they are allowed to collaboratively struggle to solve problems before they are explicitly taught the solution. To investigate whether also younger students benefit from Productive Failure, Claudia Mazziotti and colleagues investigated whether problem-solving prior to as compared to after instruction leads to more understanding. Furthermore, they examined what role students’ collaboration played by comparing collaborative problem-solving with individual problem-solving. They found no advantage of Productive Failure for this age group but found first hints for the benefits of small groups’ collaboration. These data suggest that the extent to which students are capable to deal with the initial struggle and to collaborate might display boundary conditions for Productive Failure.

## Introduction

According to the so-called Productive Failure (PF) approach^1–8^ or the Invention approach,^9–11^ students who solve problems before receiving instruction acquire more conceptual knowledge, than students who first receive instruction and then solve problems (so-called direct instruction approach, DI). Conceptual knowledge is defined as an understanding of the underlying principles and structures of a domain^12^ (e.g., why a fraction of 1/5 is smaller than a fraction of ½, even though 2 is smaller than 5). The effectiveness of these approaches has been demonstrated across different domains and samples. PF and Invention approaches resemble each other in terms of the order of the learning phases: problem solving first, followed by instruction. However, they differ in the implementation of the learning phases: Invention approaches include contrasting cases in the problem-solving phase, while PF includes typical student solutions in the instruction phase.^13^

In the following, we focus on the PF approach. During the initial problem-solving phase of PF, students usually work in small groups. Together they try to solve a yet unknown and complex problem. The complexity of a problem lies in the interaction between characteristics of the problem and of the learner (see also^6^). The characteristic of the learner that has been focused most often is students’ prior knowledge. In PF, students have not yet been formally introduced to the target concepts, but have already developed an initial understanding about pre-concepts (cf.^3,7^). This means that they do not have sufficient prior knowledge to successfully solve the problem at hand, and therefore generate incomplete and erroneous solution ideas. Regarding characteristics of the problem, in PF, the given problem allows students to generate and evaluate *multiple* solution ideas. In the subsequent instruction phase, the instructor builds upon students’ incomplete solution ideas by comparing and contrasting them with each other and with the canonical solution (i.e., instructor-led comparing and contrasting activity).^7^ Mainly three interconnected learning mechanisms are discussed to explain the effectiveness of the PF approach:^6,13^ during problem solving, students activate and differentiate their prior knowledge (mechanism 1). Across both phases, students become aware of their knowledge gaps (mechanism 2) and recognize deep features of the target domain (mechanism 3).^13^

Overall, PF is well investigated with high school, middle school, and university students typically ranging in age from 13 to 19 years. However, little is known about the effectiveness of PF for younger students around elementary school age (typically ranging from 6 to 12 years).

Some studies have implemented designs similar to PF with samples of elementary school age. These studies are comparable to PF in terms of the delayed timing of instruction^14–17^ (i.e., the core design component of PF), the overall structure of the instructional design (problem solving prior to instruction), the typical control condition (problem solving after instruction), and the learning outcome measures (i.e., conceptual knowledge). The findings from these studies regarding young students’ conceptual knowledge acquisition are mixed: only one of the studies found a superior effect of delayed instruction.^14^ Elementary school students who solved mathematical equivalence problems prior to instruction outperformed their counterparts who solved problems after instruction in terms of conceptual knowledge. However, this finding was not replicated in the other aforementioned studies^15–17^ with younger samples. Despite the commonalities between PF studies with older students and studies in younger students employing designs similar to PF, we identified two core design differences that might explain why the young students did not consistently benefit from problem solving prior to instruction: the form of instruction during the instruction phase (i.e., without a comparing and contrasting activity), and the social form of learning during the problem-solving phase (i.e., individual rather than collaborative problem solving). Our overarching research question is thus as follows: does the beneficial effect of delaying instruction after problem-solving transfer to younger students if further elements of a *typical* PF design are employed?

Form of instruction—comparing and contrasting activity: while a key design component of the instruction phase of PF lies in instructor-led comparing and contrasting between incomplete student-generated solution ideas and the canonical solution, previous studies in young students with designs similar to PF did not employ such an activity. However, it is known that instruction with comparing and contrasting activity leads to higher student learning outcomes than instruction without such activity.^7^ In a recent review, Loibl and colleagues^13^ highlighted that problem solving prior to instruction is only effective if it allows for comparing and contrasting of different solutions. The authors argued that such activity probably triggers students’ awareness of knowledge gaps and helps them to recognize and focus on deep features of the target domain (cf. PF learning mechanisms 2 and 3).

Due to their lower cognitive capacities,^18^ it might be more challenging for younger students to focus on and pay attention to these relevant deep features without support. Therefore, the comparing and contrasting activity might be particularly important for these students.

In the present study, we therefore employ an experimental design that implements an instructor-led comparing and contrasting activity, and examine the effect of the (delayed) timing of instruction (i.e. PF vs. DI) with a sample of young students under these “improved conditions”. More precisely, we hypothesized that problem solving *prior to* instruction with an instructor-led comparing and contrasting activity (i.e., PF) would lead to higher conceptual knowledge acquisition by young students, as compared with problem solving *after* instruction with an instructor-led comparing and contrasting activity (Hypothesis 1).

Social form of learning—collaborative and individual learning during problem solving: the second core difference between PF-type studies conducted with younger students and those with older students concerns the social form of learning during the initial problem-solving phase. In contrast to the majority of PF studies with older students, young students in the aforementioned studies did not try to solve a problem in small groups, but instead worked individually (or with the experimenter). Although this has not yet been investigated systematically in the PF literature, students’ collaboration is regarded as a crucial PF component,^6^ as it is meant to facilitate students’ prior knowledge activation and differentiation (cf. PF learning mechanism 1). During collaborative problem solving, students stimulate each other’s solution-generation process by mutually explaining, discussing, and questioning different solution ideas and their constraints and affordances. Therefore, groups are likely to generate more solution ideas than individuals. Research has shown that, the collaborative generation and discussion of solution ideas triggers elaborative processes^19^ such as the production of (self-) explanations and sense-making activities.^20,21^ Both groups of processes are known to support students’ conceptual knowledge acquisition (via constructive and interactive interaction with the learning material).

Against this background, in the present study, we further set out to investigate the effects of the *social form of learning* (individual vs. collaborative) during the problem-solving phase in *PF*. We expected to find higher conceptual learning in students in a collaborative PF setting as compared with those in an individual PF setting (Hypothesis 2). In addition, we assumed that students in a collaborative learning setting would generate a higher number of solution ideas during the initial problem-solving phase of PF than those in an individual setting (Hypothesis 3).

In the present study, to test the aforementioned hypotheses, we conducted a quasi-experimental study with a 2 × 2 design, varying the factors timing of instruction (i.e., problem solving prior to instruction, PF vs. problem solving after instruction, DI) and the social form of learning during problem solving (collaborative learning vs. individual learning). This resulted in the following four experimental conditions: PF-Coll, PF-Ind, DI-Coll, and DI-Ind. The instruction included an instructor-led comparing and contrasting activity and was identical across all conditions. Students learned about equivalent fractions, and were asked about their prior prerequisite mathematical knowledge in the pretest and their conceptual knowledge about equivalent fractions in the posttest (see Methods section for more details).

## Results

### Conceptual knowledge posttest score and number of solution ideas

There were no differences between conditions regarding students’ prior prerequisite knowledge (pretest) (*F*[3, 224] = 1.01, *p* = 0.39). Table 1 shows the means and standard deviations of students’ pretest and posttest scores (conceptual knowledge). A pretest score ranging from approximately four to five points demonstrates students had sufficient prerequisite prior knowledge to engage with the learning material. To test Hypotheses 1 (PF > DI for conceptual knowledge) and 2 (PF-Coll > PF-Ind for conceptual knowledge), we conducted a two-factor analysis of variance (ANOVA) with the timing of instruction (PF vs. DI) and the social form of learning (collaborative vs. individual) as factors and the posttest score as dependent variable. No significant effects emerged, neither for the timing of instruction (*F*[1, 224] = 1.01, *p* = 0.32, *η*_*p*_² = 0.005, PF: 95% confidence interval (CI) [9.75, 11.5] and DI: 95% CI [9.78, 11.91]) nor for the social form of learning (*F*[1, 224] = 3.36, *p* = 0.06, *η*_*p*_² = 0.016, Coll: 95% CI [9.75, 11.24] and Ind: 95% CI [10.11, 11.91]) nor for their interaction, (*F*[1, 224] = 0.18, *p* = 0.67, *η*_*p*_² = 0.001). The high overlap of CIs for the timing of instruction (PF and DI), the low effect size, and the statistically nonsignificant effect indicate that there was no advantage of problem solving prior to instruction. Hypothesis 1 therefore had to be rejected. To test Hypothesis 2, we calculated an a-priori contrast comparing PF-Coll to PF-Ind. In contrast to our hypothesis, there was no significant benefit of working collaboratively as compared with working individually during the problem-solving phase of PF (*F*[1, 224] = 1.16, *p* = 0.28, *η*_*p*_² = 0.005, PF-Coll: 95% CI [9.75, 10.89] and PF-Ind: 95% CI [10.11, 11.50]). Thus, Hypothesis 2 also had to be rejected.

**Table 1:** Prerequisite prior knowledge at pretest and target conceptual knowledge at posttest

	Prerequisite prior knowledge at pretest		Conceptual knowledge at posttest scores		
	M	SD	M	SD	*N*
PF-Coll	4.19	2.48	10.31	2.45	74
PF-Ind	4.00	2.04	10.80	2.41	49
DI-Coll	4.74	2.25	10.50	2.73	57
DI-Ind	4.40	2.46	11.28	2.15	48

Hypothesis 3 predicted a positive effect of collaboration (vs. individual learning) on the number of student-generated solution ideas in PF. To test this hypothesis, we conducted a one-factor ANOVA with the factor condition (PF-Coll vs. PF-Ind) and the number of solution ideas (Table 2) as dependent variable. The number of solution ideas was a dyad-level variable (*n* = 34 dyads) in the PF-Coll condition and an individual-level variable (*n* = 49) in the PF-Ind condition. No statistically significant effect emerged (*F*[1, 81] = 0.949, *p* = 0.333, *η*_*p*_² = 0.012, PF-Coll: 95% CI [3.90, 5.16] and PF-Ind: 95% CI [3.57, 4.67]). Hypothesis 3 was therefore also rejected.

**Table 2:** Number of student-generated solution ideas of the PF-Coll and PF-Ind conditions

	Number of solution ideas		Sample size
	M	SD	*N*
PF-Coll (per dyad)	4.53	1.8	34
PF-Ind (per individual)	4.12	1.92	49
Total (cases)	4.36	1.85	83

### Exploring students’ collaborative learning processes in the PF condition

Despite adapting problem solving prior to instruction in line with typical PF studies (i.e., instruction with comparing and contrasting activity and students’ collaboration during problem solving in PF), we had to reject the hypothesis that PF would lead to more conceptual knowledge than DI (Hypothesis 1), and the hypotheses that collaborative learning during PF would lead to higher benefits than individual learning during PF (Hypotheses 2 and 3). In particular, the rejection of Hypotheses 2 and 3 raises the question whether the PF-Coll students collaborated in a way that would harness the potential of the collaboration. It is clear from the literature on collaborative learning that students’ learning depends on the actually occurring interactions and not on the social form of the learning set-up per se.^22^ As the role of collaboration in PF has not yet been investigated extensively, we explored students’ collaborative learning processes in the PF-Coll condition in more detail than the above-reported quantitative analyses could reveal. More precisely, we explored how specific collaborative processes relate to students’ conceptual knowledge (on the individual level) and to the number of student solution ideas (on the dyadic level, as they were collaboratively generated). To analyze the collaborative learning processes, which are related to elaboration and sense-making activities, we built upon the Interactive–Constructive–Active–Passive (ICAP) framework by Chi and colleagues.^23,24^ The ICAP framework can serve as a tool for aligning different kinds of students’ overt learning activities (e.g., passively listing to a peer, actively reading the task, constructively engaging with the learning materials by drawing inferences or generating solution ideas, or interactively co-constructing elaborations, concepts, or creating solution ideas) to underlying cognitive processes (e.g., actively paying attention, constructively creating an explanation, and interactively co-creating a solution idea). It is hypothesized that higher levels of engagement (with passive being the lowest and interactive the highest level) are more conducive to learning, as they are more likely to involve deeper cognitive engagement with the learning material.

Accordingly, we coded students’ verbal utterances, taken from transcripts of the recorded dialogs in the PF-Coll condition. Overall, students exchanged 5074 utterances, coded as either interactive, constructive, active, coordinative, back channeling, or off-task according to our coding scheme (see Table 3 and the Methods section). Individual students produced on average 74.62 utterances (SD = 31.26). We examined how these kinds of utterances were linked to students’ conceptual knowledge and the number of student-generated solution ideas. Because according to the ICAP framework, constructive utterances (i.e., individual contributions without building upon a learning partner’s contribution)^23,24^ and interactive utterances (i.e., collaborative contributions with building upon a learning partner’s contribution) indicate deep levels of student engangement (i.e., creating processes), we were particularly interested in these types of utterances and how they relate to the number of solution ideas and students’ conceptual knowledge. In this context, we took into account both the absolute and the relative frequencies of the respective kinds of utterance. The absolute frequencies of constructive and interactive utterances represent important learning opportunities as they contain content-related contributions that go beyond the presented material paving the way for individual conceptual knowledge acquisition. The relative frequencies of these utterances demonstrate the efficiency with which students used their time for talking about relevant concepts, and thus the general quality of their collaborative problem-solving discourse.

**Table 3:** Means and standard deviations of the absolute frequencies of students’ collaborative utterances in the PF-Coll condition, measured at the individual level (*n* = 68)

	M	SD
Interactive	07.50	05.88
Constructive	14.25	06.53
Active	03.77	03.32
Coordinative	23.91	11.44
Back channeling	12.21	09.15
Off-task	12.99	12.09
Total utterances	74.62	31.26

### Student utterances and number of solution ideas

Correlation analyses (Pearson’s *r*) revealed a statistically significant positive correlation between the *absolute number* of constructive utterances (i.e., sum of constructive utterances of both dyad members) and the number of solution ideas students collaboratively generated (both variables are on the dyad level); *r*(32) = 0.411, *p* = 0.008). That is, the more constructive dyad members were, the more solution ideas a dyad generated. All other types of utterances were statistically not significantly associated with the number of solution ideas (interactive: *r*(32) = 0.251, *p* = 0.076; active: *r*(32) = 0.084, *p* = 0.319; coordinative: *r*(32) = 0.285, *p* = 0.051; back channeling: *r*(32) = 0.214, *p* = 0.0113, off-task: *r*(32) = 0.076, *p* = 0.335). Regarding the *relative number* of utterances (i.e., sum of the respective kind of utterances a dyad exchanged divided by the dyad’s total number of utterances), we found no significant correlations between the number of solution ideas and the relative number of interactive (*r*(32) = 0.074, *p* = 0.338); constructive (*r*(32) = 0.066, *p* *=* 0.355), active (*r*(32) = –0.044, *p* = 0.403), coordinative (*r*(32) = 0.035, *p* = 0.423), back channeling (*r*(32) = 0.064, *p* = 0.360), and off-task utterances (*r*(32) = –0.125, *p* = 0.241; all variables are on the dyad level).

### Student utterances and conceptual knowledge at posttest

Correlation analyses revealed no significant correlations between any *absolute frequency* of utterances (i.e., sum of the respective kind of utterances of an individual) and students’ conceptual knowledge at posttest (interactive: *r*(66) = 0.18, *p* = 0.15, constructive: *r*(66) = 0.16, *p* = 0.19, active: *r*(66) = 0.05, *p* = 0.71, coordinative: *r*(66) = –0.19, *p* = 0.12, back channeling: *r*(66) = –0.11, *p* = 0.37, off-task: *r*(66) = –0.23, *p* = 0.06; all variables are on the individual level). The correlation between the *relative frequency* of student utterances (i.e., sum of the respective kind of utterances an individual made divided by the total number of utterances the individual contributed) and posttest showed a different pattern. The relative number of interactive (*r*(66) = 0.219, *p* = 0.036), constructive (*r*(66) = 0.233, *p* = 0.028), and active (*r*(66) = 0.226, *p* = 0.032) utterances correlated significantly with students’ conceptual knowledge at posttest (note that all variables are on the individual level). As predicted by ICAP, the more interactive, constructive, and active a student was (i.e., relative frequencies), the higher he/she scored in the conceptual knowledge posttest. There were no significant correlations between the relative number of any other type of utterances and students’ conceptual knowledge (coordinative: *r*(66) = –0.091; back channeling: *r*(66) = –0.135, *p* = 0.136; off-task: *r*(66) = –0.171, *p* = 0.082; all variables are on the individual level).

Overall, our additional process analyses revealed two main findings: first, the more dyad members were constructive (absolute frequency), the more solution ideas the dyad generated or in other words the more learning opportunities the dyad encountered. Second, the more interactive, constructive and active an individual student was (relative frequency), the more conceptual knowledge the student acquired. We further elaborate on these findings in the respective Discussion section.

## Discussion

PF and similar learning approaches have repeatedly demonstrated their effectiveness for students’ conceptual knowledge acquisition in samples of adolescents and young adults. Previous studies in children around the elementary school age, which did not employ PF, but implemented designs sharing the characteristic element of delayed instruction with PF,^14–17^ yielded mixed results regarding the effectiveness of delayed timing of instruction on students’ conceptual knowledge. We identified two core design differences between the two types of studies, which might explain why younger students, in contrast to older students, did not consistently benefit from problem solving prior to instruction. The first difference refers to the absence of a comparing and contrasting activity during instruction and the second difference relates to students’ collaboration during problem solving. Both design components were typically implemented in PF studies with older students, and are thought to trigger the aforementioned learning mechanisms underlying the effectiveness of PF. In the present study, young students around the elementary school age learned with a typical PF design. Our learners collaboratively engaged in problem solving prior to receiving instruction, and this instruction included an instructor-led comparing and contrasting activity. Despite having adapted the implementation to align with previous PF studies, we were unable to replicate the beneficial effect of delaying instruction until after problem solving with young students. Thus, we had to reject Hypothesis 1 (PF > DI for conceptual knowledge). Furthermore, we found no beneficial effects of collaborating in small groups (vs. working alone) in PF. Students in the collaborative condition neither acquired more conceptual knowledge (Hypothesis 2) nor generated more solution ideas during the initial problem-solving phase (Hypothesis 3) than students in our individual PF condition.

Regarding the three main hypotheses, our study revealed null results, even though its statistical power was adequate to detect at least medium-sized effects. The challenge of null results lies in their interpretation. On the other hand, the null results could originate from flaws in our study design. First, the design of the PF learning material could be criticized for being too easy or too hard. Because the design of the material was strongly driven by and iteratively improved according to PF design recommendations^6^ and our students had sufficient prerequisite prior knowledge to be able to interact with the learning material (see pretest score in Result section), we believe, however, that the learning materials, and our implementation of PF in general, cannot explain the null effects. Another point of criticism in our design could refer to the conceptual knowledge posttest. While the test’s validity can be considered to be high as it was closely designed in consultation with subject matter experts, its internal consistency was low. The low internal consistency could relate to the heterogeneous nature of the posttest (i.e., assessing different target concepts) and the way it interacted with the setting of our study. In a real classroom setting, the allotted time for the posttest and thus the number of posttest items we were able to pose to the children, was rather limited. This presumably contributed to the relatively low internal consistency of the test. More test items (for each target concept) could have increased the internal consistency of the test. However, as we found no indication of statistical differences between experimental conditions, we doubt that differences could have been found even with a test of higher internal consistency. On the other hand, the null results might indicate that there really is no advantage of PF over DI for this young sample. Support for this interpretation lies in the small effect sizes and highly overlapping CIs between problem solving prior to, as compared with after instruction (i.e., PF vs. DI). These patterns were also found in a similar study by Chase and Klahr.^25^ Their study and our study, thus, add to the number of problem solving prior to instruction studies in students around the elementary school age, which showed no clear advantage of problem solving prior to instruction over problem solving after instruction.^15–17,25^ Despite the aforementioned limitations, we still believe our study makes a relevant contribution to the PF literature and leads to the question whether younger children might lack crucial prerequisites to *productively* learn from PF and similar approaches. But until having conducted PF studies that systematically compare different age groups, we cannot know whether students’ age, or another factor, explains the null results in our and other studies. This poses an experimental dilemma. While, for example, the most “popular” PF problem (involving standard deviation), is appropriate for adolescents and young adults, it is far too hard for young students around the elementary school age, because they do not have the prerequisite prior domain-specific knowledge to meaningfully engage with the problem. Problems that are appropriate for elementary school students, in turn, do not provide failure opportunities for older students, as they have already learnt the canonical solution for these problems in school. In other words, the PF effect cannot materialize across both age groups when using the exact same learning material. Nevertheless, with the aim of contributing to future research hypotheses, we would like to open the discussion about potential characteristics of young learners that might limit their ability to benefit from PF.

Potential cognitive limitations of younger students: first of all, young students might not have sufficiently developed the necessary cognitive capacities for benefiting from PF. During unsupported problem solving, the cognitive demand tends to be high as previous PF research^4^ demonstrated. For example, learners need to break down the problem, monitor problem-solving steps, and keep track of goals and sub-goals. This requires working-memory capacity, particularly executive functions. However, the executive functions of children around the elementary school age, and thus their ability to regulate their actions and monitor problem-solving steps, are not as developed as the ones of older students.^18^ Therefore, the beneficial effect of delaying instruction after problem solving might not transfer to younger students due to their comparably lower general cognitive capacities (as discussed by Loehr et al.^16^). Future research is needed to better understand how young students’ cognitive capacities, and learning outcome relate to one another in PF and similar settings.

Moreover, in contrast to older students, younger students are likely to have less advanced self-regulatory learning strategies^26^ such as dealing with failure and controlling for motivation, attention, and emotions.^27^ These strategies, however, might be an important prerequisite for persistently engaging in solving a problem that is designed to be challenging (and can thus be frustrating) and to generate *multiple* (but not necessarily complete) solution ideas. To generate multiple solution ideas is important because it is discussed to relate to students’ prior knowledge activation and differentiation^3,6,13^ (cf. learning mechanism 1). In our study, however, the PF students generated a rather low number of solution ideas. On average they generated only 4.36 (SD = 1.85) solution ideas out of the range of possible solution ideas (e.g., calculating with fractions, using concrete or abstract graphical representations, trying out equivalents of fractions, or making use of more creative approaches, see Methods section). This result supports the notion of young students’ low(er) persistence in regulating their own problem solving. To shed more light on the potential role of young students’ self-regulation strategies for the effectiveness of PF, again future research is needed.

The role of collaboration: in our sample of children around the elementary school age, we found no benefits of working collaboratively vs. individually during PF. Our additional process analyses, however, revealed two main findings that might inform the design of PF interventions that are productive for young students. First, the more dyad members engaged in constructive utterances (e.g., by posing questions, generating analogies, or drawing inferences to go beyond the information presented in the original problem^24^), the more solution ideas the dyad generated. These solution ideas, in turn, represent important learning opportunities for the students. To facilitate dialogs with a high number of constructive utterances, a potential implication for future PF studies could thus be to prompt students’ collaboration. In addition to provide students with the PF typical motivational prompts during problem solving (as in our study), we would thus suggest to design prompts that specifically target students’ *constructive* contributions.

Second, students who showed high relative levels of constructive and interactive utterances in the discussion with their partner scored higher in the conceptual knowledge posttest. This result is in line with the ICAP framework and with broader research on collaborative learning: both constructive and interactive utterances require creating, sense-making, and elaboration processes, which are known to facilitate students’ conceptual knowledge acquisition.^19–21^ A similar pattern was found for the relative levels of active utterances, which according to the ICAP taxonomy trigger a lower but still beneficial level of students’ cognitive engagement (i.e., attention processes rather than creating processes). Thus, higher individual conceptual knowledge is associated with higher quality of individual talk during problem solving (with causality probably present in both directions). Regarding possible support for younger students in PF, this mainly points towards one precondition, namely students’ ability to engage in high-quality dialog (i.e., with high levels of at least active, or even constructive and interactive utterances). In line with German mathematical standards, our young students should have met this precondition and should have been able to communicate and to engage in mathematical reasoning.^28^ However, younger students might still need additional practice with engaging in learning-centered dialog with peers before they can benefit from collaboratively tackling problems in a PF setting, especially as it takes time (and practice) for the potential of collaborative learning to unfold.^29^ Thus, future PF research should continue to investigate the role of the students' experience in small group collaboration in the effectiveness of PF. One option would be to observe students and their collaboration in longitudinal research designs.

Analyzing collaborative engagement in PF: to analyze students’ collaborative processes in the PF-Coll condition, we mainly drew upon the ICAP framework. This framework relates different kinds of overt learning processes to underlying cognitive processes, resulting in a proposed hierarchy of learning processes (I > C > A > P). *Interactive processes* are said to be more beneficial for students’ learning than *constructive* processes, as they trigger *joint* creating processes by interactively building on a learning partner’s contribution rather than creating processes by constructively giving a (self-) explanation. Both processes are said to be more beneficial than *active* (e.g., reading parts of the problem, triggering attending processes) and *passive* processes (e.g., engaging physically with learning material). The ICAP framework, thus, covers a variety of different learning processes that can be expected to occur during students’ collaborative problem solving (in PF). The mode of engagement that the ICAP framework terms *interactive* synthesizes beneficial collaborative processes that have been described by a large body of previous research. Such process features include students’ joint focus of attention,^30^ flow of collaboration,^31^ or transactive interactions.^32^ Following the ICAP framework’s prediction, interactive processes should show the strongest relationship with learning. However, we found no clear superiority of interactive processes in our study. Regarding the generation of solution ideas, the number of constructive utterances was not only higher (constructive: M = 14.25, SD = 6.53 > interactive M = 7.50, SD = 5.88) but also more relevant than the number of interactive utterances. During the PF phase, the cumulative contributions of individual students, rather than the co-constructed ideas, determined how many solution ideas a dyad noted down. Regarding the individual posttest score, the relative numbers of interactive, constructive, and active utterances all showed a positive relationship. Against this background, it seems that the most relevant factor for learning is content-related talk (and not the social mode in which it was generated). This major effect might have overshadowed the differential effects of the different types of utterances (i.e., constructive and interactive utterances). The missing evolvement of the ICAP hierarchy (I > C > A > P) might relate to two aspects: first, the categories of the ICAP framework (which was primarily meant to guide the *design* of learning environments and not the analysis of dialog patterns in a specific learning environment) may be too broad to capture different nuances of collaborative learning such as giving explanations, posing questions, justifying claims and so on. Second, the overt learning processes might not always reveal the underlying cognitive learning processes, especially when differentiating between constructive and interactive processes. Even though the theoretical and coded differentiation between interactive and constructive activities seems clear, the point when a constructive contributions becomes an interactive contribution might not always be visible.^33^ Does no explicit reference to a previous contribution automatically mean that students did not interactively build on this previous contribution and did not integrate the previous idea? This unclear relation between the overt learning activity and the cognitive learning processes may explain the missing superiority of interactive utterances in our study. To shed more light on this relation in particular and on the role of collaboration in PF, more in-depths analyses are needed.

In summary, our study failed to demonstrate the often beneficial effects of PF in a sample of younger students but opened the stage for discussing potential boundary conditions of PF relating to students’ age. However, these boundary conditions need to be investigated and validated in future studies.

## Methods

### Sample

A total of 228 German fifth graders (aged 10–12 years) from nine classes from three different schools participated in the study. Classes (approximately 22–30 students per class) were randomly assigned to conditions (i.e., PF-Coll: three classes, PF-Ind: two classes; DI-Coll: two classes; DI-Ind: two classes, see also Table 1). As in previous PF studies^3,7^ and in line with the German core curriculum,^28^ students did not have any formal knowledge about the target domain. In all classes, parental consent for students’ involvement was obtained.

### Learning material

**Fig. 1:**
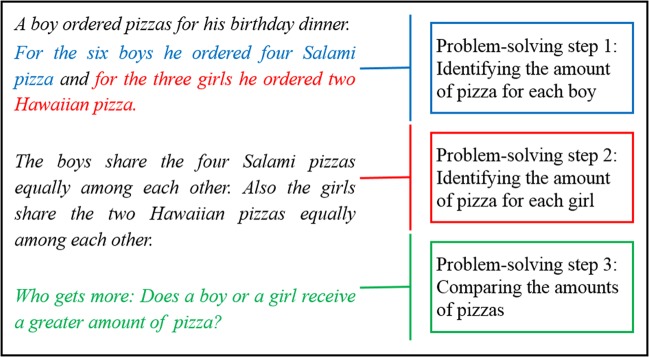
Equivalent fraction problem used in both Productive Failure conditions. In the direct instruction conditions, students had to solve an isomorphic problem differing only in the numbers being used in problem-solving step 1 and 2 (i.e., the boy ordered two Salami pizza for four boys and four Hawaiian pizza for eight girls)

Students acquired conceptual knowledge about fraction equivalence comprising three interconnected target concepts. The first concept (relation) required students to understand the relation between the size and the number of fractional parts within a given fraction (i.e., the more slices a pizza is cut into, the smaller the size of each individual slice will be). The second concept (division) concerned the division of fractions. It required students to understand to divide, for example, a circle representation through the central point and to fairly distribute these parts among persons. The third concept (comparison) focused on the comparison across two given fractions for identifying fraction equivalence. To be able to compare two fractions with different denominators and numerators, students needed to understand the *necessity* to first find a common denominator (prior to comparing). This way students were able to “mathematically argue” for the existence or absence of equivalence. For example, when students found a common denominator, they were able to “mathematically argue” that a person who receives 3/6 of a pizza has the same amount of pizza as a person who receives 6/12, although 6/12 has a greater numerator and denominator (representing a common preconception of students at the transition between grades 5 and 6^34^). According to the German standards of the core curriculum, arguing with mathematical concepts is a key competence that students should have acquired at the latest by the end of grade 4.^35^ The learning material included a cover story on sharing pizzas (see Fig. 1).

### Problem-solving phase within PF and DI

Across all experimental conditions, the problem required students to divide a given number of pizzas of one type (“salami”) between a number of boys (problem-solving step 1) and a different number of pizzas of a second type (“Hawaiian”) between a number of girls (problem-solving step 2); finally, they had to compare and conclude whether boys and girls received the same amount of pizza each (problem-solving step 3). The problem description did not make the required problem-solving steps salient and did not pre-structure students’ problem-solving process.

As students had no formal target knowledge, PF students encountered a rather complex problem-solving situation, which is an important and suitable design component for PF^6^ as described in the Introduction. Further, and also in line with these design recommendations, the problem permitted students to develop multiple solution ideas. For example, they could try to find a solution by calculating with actual fractions (e.g., using valid or invalid strategies for generating and comparing fractions); by making inferences based on graphical representations of the problem (e.g., some students drew the two types of pizzas and “cut” them into slices of equal or unequal size); by using more abstract graphical representations of fractions (e.g., circles divided into halves, quarters, and other equivalents); or by applying a more creative strategy (e.g., one dyad argued that the “birthday boy” should receive a whole pizza to himself, which would alter the whole problem). Students in the PF conditions tried to solve the problem shown in Fig. 1 and were asked to come up with as many solution ideas as possible. To prevent frustration while working on a challenging problem, the instructor provided PF students with motivational prompts (e.g., you are doing fine, keep going) during problem solving.

In the DI condition, the problem shown in Fig. 1 was part of the instruction. Thus, DI students received an isomorphic problem with different numbers for each problem-solving step (i.e., each boy received 2/4 of a pizza and each girl received 4/8 of a pizza, again resulting in an equal amount of pizza for each child). As the problem-solving phase in DI only occurred after the instruction, students were not expected to generate multiple solution ideas.

### Instruction phase in PF and DI

The instruction was constant across all four experimental conditions. It used the problem employed in the problem-solving phase of the PF conditions, and included comparing and contrasting between student-generated solution attempts and the canonical solution. A table with more details on the comparing and contrasting activity is presented in the supplementary material. We employed typical student solutions taken from two previous studies^36^ rather than the solutions of the students in this study. This enabled us to keep the instruction constant and it prevented a variety of different and unpredictable solutions across experimental conditions, which would have led to unwanted variance.

The instruction also allowed students to practice the solution steps required to solve the types of problem the PF students already had encountered during the initial problem-solving phase of PF or likewise the DI students still had to encounter during the subsequent problem-solving phase of DI.

Students practiced to divide and compare fractions on worksheets.

### Procedure

The study was conducted during students’ regular mathematics lessons. All participants answered the pretest (10–15 min) in the mathematics lesson preceding the study. During the intervention lessons, all students received a 10- to 15-min introduction about the study background. Afterwards, students in the PF conditions started with the problem-solving phase and then received instruction. Students in the DI conditions first received instruction and then engaged in the problem-solving phase. Regardless of condition, the problem-solving phase took approximately 30 min and the instruction approximately 45 min. After they had completed both learning phases, students had 45 min to individually work on the conceptual knowledge posttest.

### Knowledge tests

#### Pretest

In line with previous PF studies,^7^ the pretest did not measure the targeted knowledge (which students could not be assumed to already possess), but rather prerequisite mathematical knowledge. This ensured that students were not prompted to generate solution ideas prior to receiving instruction.^7^ The pretest tested mathematical prerequisites such as naming fractions, dividing natural numbers with and without remainder, and understanding fractions as part of a whole. There were a total of nine questions, from which students could receive a maximum of 9 points. As the pretest measured prerequisite prior knowledge rather than the target knowledge, it did not correlate with students’ conceptual knowledge at posttest (*r*(226) = 0.01, *p* = 0.15) and was thus not included in further analyses.

#### Posttest

**Fig. 2:**
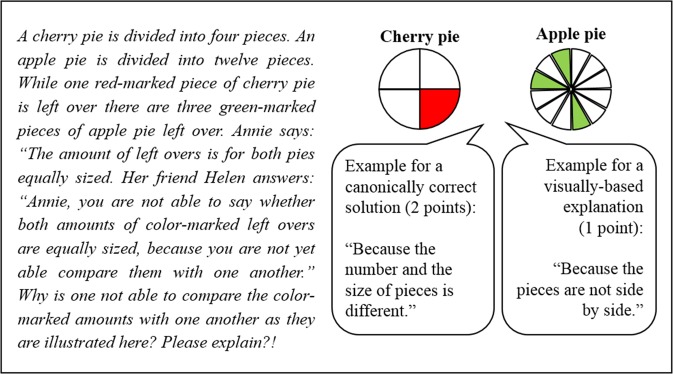
Example item of the conceptual knowledge posttest used across all conditions

The conceptual knowledge posttest was designed with the aim of testing for a deep understanding of fraction equivalence and of resembling a “regular” mathematics exam. Therefore, it included non-trivial tasks that required students to solve story problems of similar complexity to the problem, which they had encountered during the learning phases and was limited in time to one regular 45-min school lesson). The posttest comprised six items: one multiple-choice item including a drawing task, and five story problems (with subtasks) that required explanation-based answers. To code students’ responses to the story problem items, we distinguished between two categories of explanations (see also Fig. 2): the canonically correct explanation (e.g., comparing two fractions by expanding or reducing one of the fractions to get the same denominator for both fractions) and an explanation based on a visual strategy (e.g., comparing two fractions by visually putting smaller pieces of fraction A together to get the same size of one piece of fraction B). We rewarded the canonically correct explanation with 2 points, the visual-based explanation with 1 point, and any other (or no) explanation with 0 points. For the multiple-choice item, students were rewarded with 1 point for the correct answer and an additional point if they were able to correctly visualize their answer. The maximum total possible score was 18. Cronbach’s alpha was *α* = 0.54 across the six items (*n* = 228). Given the heterogeneous nature (i.e., assessing different target concepts, see Learning material section) and the shortness of our posttest, we considered this an acceptable level of consistency.

### Process analyses

#### Number of solution ideas

To investigate whether the *number of solution ideas* differs across both PF conditions (cf. Hypothesis 3) and how it relates to different types of students’ utterances (cf. collaborative process analyses), we coded and analyzed th*e number of solution idea*s students developed for each problem-solving step rather than the number of complete solutions (i.e., students attempt to engage in *all three* problem-solving steps). As a coding example, drawing the four salami pizzas and dividing them into halves was coded as one solution idea for problem-solving step 1, and again drawing the four salami pizzas and this time dividing them into quarters was coded as another solution idea for problem-solving step 1. We summed up the number of solution ideas across all problem-solving steps into one sum score. Two coders were intensively trained to assess the number of solution ideas. To determine interrater reliability for the number of solution ideas, the raters both coded 43% of the solution sheets (*n* = 35 cases across both PF conditions), with satisfactory interrater reliability (number of solution ideas κ = 0.65). Disagreements were resolved by discussion.

#### Analyzing different types of collaborative utterances

To assess different types of collaborative utterances and how they are linked to students’ conceptual knowledge and the number of solution ideas, we analyzed students’ verbal utterances, which had been audiotaped during the collaborative problem-solving phase. We transcribed the audiotapes of all dyads of the PF-Coll condition (*n* = 34 dyads; audio data for three dyads were lost due to technical failure). We developed and iteratively improved a coding scheme by following a top-down and bottom-up approach:^37^ using the bottom-up approach, the coding scheme builds on verbal process data from a previous study^36^ in which dyads tried to solve the very same equivalent fraction problem prior to receiving instruction (see Fig. 1). From the top-down perspective, the coding scheme is rooted in the ICAP framework^23,24^ and broader literature about collaborative communication processes.^38^

To avoid ambiguity of interpretation and thus to increase objectivity, we coded on the utterance level rather than on an episode level. The final coding scheme differentiates between six different types of collaborative utterances: interactive, constructive, active, coordinative, back channeling, and off-task. While interactive, constructive, and active utterances are derived from the ICAP framework, coordinative, back channeling, and off-task utterances are taken from the broader literature on collaborative learning. Table 4 presents the conditions that needed to be fulfilled to code a single utterance as interactive, constructive, active, coordinative, back channeling, or off-task. Overall, we used a conservative coding approach, choosing the lower code in cases of ambiguity (i.e., the one indicating lower engagement). Two coders were intensively trained on the coding scheme. Interrater agreement was tested with a subset of 15% of randomly selected transcripts (*n* = 5 transcripts) and was high (ICC_absolute_ = 0.92; 95% CI [0.49, 0.96]). Inconsistencies were resolved by discussion.

**Table 4:** Overview of coding scheme with coding examples

Codes	Conditions for labeling an utterance as	Examples (translated by the author)
Interactive	• Builds upon a prior contribution of the learning partner • Reflects a mathematical content • Mathematical content reflects an idea that goes beyond the information included in the equivalent fraction problem^a^	Student 1: Well, when we divide the pizza for boys into halves, then the first boy gets a half, the second gets a half, the third gets a half… Student 2: …the fourth gets a half, the fifth gets an entire and the sixth gets an entire piece.
Constructive	• Does not build upon a prior contribution of the learning partner • Reflects a mathematical content • Mathematical content reflects an idea that goes beyond the information included in the equivalent fraction problem^a^	Student 3: Well, this is six times four, four pizzas and then we can divide six by four and four…hmh, four times six are 24.
Active	• Students read aloud the equivalent fraction problem or parts of the problem	Student 2: For the six boys he orders four Salami pizza and for the three girls he orders two Hawaiian pizza, because the boys share the pizza among each other. Also the girls share the pizza among each other. Student 6: Yes, look. Who receives more? A boy or a girl receives a greater proportion of pizza.
Coordinative	• Does not reflect a mathematical content that goes beyond the information included in the equivalent fraction problem • Students coordinate their work by saying who will do or has done which aspect of the problem	Student 4: I have to, hmhm, well we have already painted but we did not calculate yet.
Back channeling	• Does not reflect any mathematical content • Reflects agreement or disagreement • Indicates the learning partner to continue to listen • Reinsures the learning partner’ understanding	Student 7: Hmhm, yes. Student 8: Did you understand? Student 7: Yes, I did.
Off-task	• Any other utterance that does not refer to the equivalent fraction problem • E.g., talking about the weather or a new haircut	Student 5: Welcome to the news.

^a^Examples for mathematical content that goes beyond the presented material are dividing, adding, multiplying fractions, or using graphical representations of fractions

To assess the number of learning opportunities students encountered during the problem-solving phase, we used the absolute frequency (i.e., sum) of different kinds of utterances. To examine how efficient students used their collaborative discourse time, we referred to the relative frequencies (i.e., sum of respective utterance divided by the total number of utterances). To investigate the relation between the number of solution ideas and the absolute frequency of different kinds of utterances, we referred to the absolute number of the respective kind of utterances a dyad made.^39^ To analyze the association between the number of solution ideas and the relative frequency, we referred to the number of the respective kind of utterance divided by the total number of utterances both dyad members exchanged (e.g., sum of interactive utterances of both dyad members divided by the total number of utterances of both dyad members). Regarding the relation between students’ conceptual knowledge and the aforementioned types of utterances, we referred to either the absolute frequency of interactive, constructive, active, coordinative, back channeling, and off-task utterances an individual student produced, or to the relative frequency, i.e., the respective utterance an individual produced divided by his/her total number of utterances (e.g., interactive utterances of an individual divided by his/her total number of utterances).

Note that the methods were performed in accordance with relevant regulations and guidelines and approved by members of the Institute of Educational Research.

### Reporting summary

Further information on experimental design is available in the Nature Research Reporting Summary linked to this article.

## Supplementary information

Supplementary Material - Table I.

Reporting Summary

## Data Availability

Due to legal data privacy issues, the data to support the findings of this study are not publicly available, as individual students might be identifiable to insiders. The anonymized datasets may be provided by the corresponding author on reasonable request.

## Supplementary information

**Supplementary information** accompanies the paper on the *npj Science of Learning* website (10.1038/s41539-019-0041-5).
