# Supplementary material for: Probing boundary conditions of Productive Failure and analyzing the role of young students’ collaboration
Source: NPJ Sci Learn. 2019 Mar 26;4:2. doi: 10.1038/s41539-019-0041-5 (PMC6435728; doi:10.1038/s41539-019-0041-5)
Supplement: Supplementary file 1 — Supplementary Material - Table I. [file 41539_2019_41_MOESM1_ESM.pdf]

Table I Comparing and contrasting activity in the instruction phase for problem-solving step 1

Examples for typical erroneous student solutions and the canonical solution	Activity of the instructor in the role of the teacher
	Step 0: Estimation question: Does a boy (and girl respectively) get more or less than an entire pizza?
Student solution 1:<br>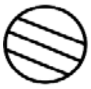	Step 1: Drawing student solution 1, counting the number of pieces and highlighting the absence of drawing through the central point
Student solution 2:<br>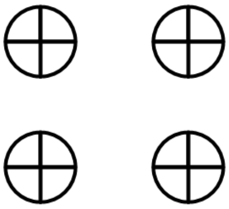	Step 2: Drawing student solution 2 and counting the number of pieces of pizza (=16)<br>Step 3: Dividing 16 pieces of pizza by 6 boys which results in 2 pieces for each boy with a remainder of 2 pieces<br>Step 4: Guiding students' attention to the goal of dividing equally and fairly which is not yet reached by cutting the pizzas into quarters<br>Step 5: Finding another way of cutting the pizza into pieces
Student solution 3:<br>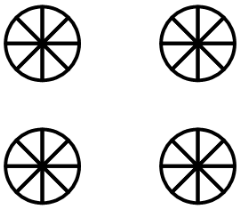	Step 6: Repeating Steps 2-5 for student solution 2<br>Step 7: Asking how the number of boys is linked to the number of pieces of pizza?
Canonical solution:<br>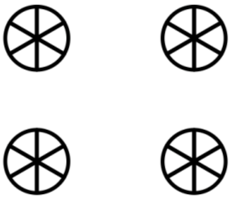	Step 8: Presenting the canonical solution by contrasting it with the previous student solutions
Answer:<br>A single boy receives 4 pieces of pizza (i.e. four sixths)<br>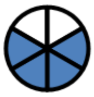	
